# A New Algorithm for Digital Image Encryption Based on Chaos Theory

**DOI:** 10.3390/e23030341

**Published:** 2021-03-13

**Authors:** Yaghoub Pourasad, Ramin Ranjbarzadeh, Abbas Mardani

**Affiliations:** 1 Department of Electrical Engineering, Urmia University of Technology, Urmia 57561-51818, Iran; 2 Department of Telecommunications Engineering, Faculty of Engineering, University of Guilan, Rasht 45371-38791, Iran; ranjbar.ramin24@gmail.com; 3 College of Business, University of South Florida, Tampa, FL 33813, USA; abbasmardani@usf.edu

**Keywords:** digital image encryption, image processing, chaos random sequence, discrete wavelet transform

## Abstract

In recent decades, image encryption, as one of the significant information security fields, has attracted many researchers and scientists. However, several studies have been performed with different methods, and novel and useful algorithms have been suggested to improve secure image encryption schemes. Nowadays, chaotic methods have been found in diverse fields, such as the design of cryptosystems and image encryption. Chaotic methods-based digital image encryptions are a novel image encryption method. This technique uses random chaos sequences for encrypting images, and it is a highly-secured and fast method for image encryption. Limited accuracy is one of the disadvantages of this technique. This paper researches the chaos sequence and wavelet transform value to find gaps. Thus, a novel technique was proposed for digital image encryption and improved previous algorithms. The technique is run in MATLAB, and a comparison is made in terms of various performance metrics such as the Number of Pixels Change Rate (NPCR), Peak Signal to Noise Ratio (PSNR), Correlation coefficient, and Unified Average Changing Intensity (UACI). The simulation and theoretical analysis indicate the proposed scheme’s effectiveness and show that this technique is a suitable choice for actual image encryption.

## 1. Introduction

In recent years, image encryption has been an attractive area for research. It is extensively recognized as a useful technique for secure transmission. Every image encryption algorithm is aimed to generate a noisy image’s top-quality to keep information secret [1,2]. Additionally, image encryption has a preferable part for guaranteeing classified transmission and image capacity over the web. Digital communication has become broader by the fast development of Internet technology [3,4]. People can send a digital image on the Internet anytime and anywhere [5,6]. This has resulted in the development of digital image encryption. Different methods representing digital image encryption in studies are connected to the ever-increasing necessity of security. Image encryption based on the chaos method is a novel encryption method for images where a random chaos sequence is applied for encrypting the image as an effective way for solving the intractable problems of highly secure and fast image encryption. Over the last few years, various versions of the chaos technique have been presented. Presently, four approaches have been adopted for image encryption, applying various principles individually and achieving the same objectives. The four principles include sharing and secret segmentation, sequential permutation, chaotic dynamical systems, and modern cryptography, each with unique features [7,8,9,10,11,12,13]. Chaos-based effective selective image encryption [14] was introduced by Khan et al. First, the plaintext image is initially divided by the proposed technique into some blocks. The correlation coefficients are determined. The block with the highest association coefficients is pixel-wise eXclusive OR-2ed (XORed) with the random numbers created from a skew tent map in terms of a pre-determined threshold value. Ultimately, the entire image is permuted through two random sequences created from Two Dimensional Ellipse Reflecting Chaotic System (TD-ERCS) chaotic maps. A novel fast image encryption algorithm oriented by chaos [15] was introduced by Wang et al., oriented by the permutation-diffusion architecture. In their method, the image is first separated into pixel blocks.

The spatiotemporal chaos is then utilized for shuffling the blocks and simultaneously changing the pixel values. Patidar et al. represented another vigorous pseudorandom permutation-substitution outline based on chaos for image encryption [16]. It was a loss-less symmetric block cipher and designed especially for color images. It may also be utilized for grayscale images. Wang et al. proposed a block image encryption outline in dynamic random growth and chaotic hybrid maps [17]. Since the cat map is simply fractured by selected plaintext attack, and it is periodic, in another securer way, they used the cat map for eliminating the cyclical phenomenon and resisting selected plaintext attack. Volos et al. presented an image encryption procedure in terms of chaotic synchronization phenomena [18]. They provided a new image encryption scheme through a chaotic TRBG (True Random Bits Generator). Image encryption based on synchronizing fractional chaotic systems [19] was utilized by Xu et al. A DNA sequence and a hybrid genetic algorithm were used for image encryption by Enayatifar et al. [20]. They presented a new image encryption algorithm using a hybrid model of a genetic algorithm (GA), deoxyribonucleic acid (DNA) masking, and a logistic map. Xu et al. introduced a novel bit-level image encryption algorithm oriented by chaotic maps [21]. Chaos-based Genetic Algorithms are extensively utilized for image encryption by many researchers [22,23,24,25,26]. Multiple-image encryption through the robust chaotic map in wavelet transform domains [27] was represented by Li et al. In this work, first, discrete wavelet transform (DWT) was used to decompose the original images being used and reassemble the lower frequency components as the direct images (estimated images). The direct image was then totally scrambled through Arnold’s cat map. Third, further decomposing the scrambled image and the resulting block images are employed separately to integrate with the amplitude parameter of the RCM (robust chaotic map) for generating keystream in each diffusion procedure. Satish et al. presented an outline to encrypt an image through the Logistic Map [28]. It would scramble the image pixels. Thus, the resulting cipher image will be XOR encrypted while dividing the output into various frequency coefficients through Integer Wavelet Decomposition. The Logistic Map is used to shuffle the resulting low-frequency coefficient wavelet, and all the frequency coefficient wavelets will be integrated via Inverse Integer Wavelet Transformation. The main objective of this manuscript provides a new technique based on chaos theory for digital image encryption. Nevertheless, the chaos-based image encryption technique has some problems, including limited accuracy. For this reason, in this research, the encryption of images is divided into spatial and transform domain encryption. Over the last few years, some image encryption schemes were presented by the frequency domain and spatial domain. Spatial domain methods directly act on the pixels of the plain image. Because this method contains high-speed encryption, it is used widely [29,30]. The transform domain encryption is used, considering some typical properties of digital images as a strong correlation between high redundancy and nearby pixels.

A method of encryption-decryption employing Rivest–Shamir–Adleman (RSA) algorithm components and topological image protection was suggested by Kovalchuk et al. The main advantages of the suggested approaches are accomplished by using images with functional fluctuation intensity [31]. The effect of the noise-adding functions added to the source picture, and also the different values of simple numbers of the RSA scheme on the effects of the process were analyzed in another work. These results were set to not give rise to the presence of contours in the encrypted image [32]. Additionally, other approaches, such as linear and quadratic fractal algorithms [33,34], projective transformations [35], and binary linear-quadratic transformations [36], are also used for image encryption and decryption. A model of image encryption based on a complex chaos-based pseudorandom number generator and modified advanced encryption standard was proposed by Hafsa et al. On the Altera Cyclone III board, the overall system was created. The findings revealed that the cryptographic algorithm was quicker and could withstand attacks of some kind [37]. A novel grayscale image cryptosystem based on chaotic hybrid maps was introduced by Kari et al. The proposed scheme has better properties, including broader chaotic ranges and more dynamic chaotic behavior, based on the results [38].

This paper is oriented by the chaos sequence and wavelet transform value and the integration of the image encryption algorithm. Such algorithms are simulated through analyzing the algorithm to discover the gaps. Thus, the algorithm was enhanced. This method uses two one-dimensional chaotic systems that can use even a fundamental nonlinear equation to display chaotic behavior. Our main aim, as well as the proportion of taking this kind of map, is to discover a new discrete-time sequence, the same as the chaotic output of the logistic map with elementary equations with unique parameters.

This paper is presented in the following sections. In the “Introduction” section, the motivation and the statement of the problem are described. Moreover, the literature review of the related papers is interpreted in this section. Furthermore, in the “Methods and Materials,” the basic mathematical concepts and expression of the proposed method are presented. Moreover, in the “Proposed Algorithm” section, the result of the proposed model implementation is described using graphical figures and tables. In addition, the comparison is presented in the “Proposed Algorithm” section. In the “Discussion” section, the findings are interpreted, and previous studies, hypotheses, limitations and suggested future works are described. Finally, the “Conclusions” section summarizes the results by numerical outcomes and perspective concepts.

## 2. Materials and Methods

### 2.1. Chaos and Transformation Theories

Nowadays, chaos and transformation theories have emerged as novel currencies in social sciences. Image transformation is a technique simplifying image processing and improving the performance of image processing. Image enhancement denotes highlighting and sharpening definite features. It includes the contours, edges, and contrast of an image to display, observe, or further analyze and process the image [39,40,41,42]. Chaos theory presents the 1st Transdisciplinary understanding of bifurcation and transformational change. As a mathematics field, it has focused on the dynamical systems’ behavior with extreme sensitivity to primary conditions. Numerous attempts exist to apply chaotic signals for communications. However, there is a lack of a useful way for recovering chaotic signals from noises larger than the signals.

### 2.2. Chaotic Sequence Based on Logistic Map

A one discrete-time-dimensional nonlinear system displaying quadratic nonlinearity is called a logistic map. The logistics map is shown with the following function. *f*: [0, 1] →ℜ as

(1) $$f\left(x\right)=\mu_{x}\left(1-x\right)$$

which is stated in state equation form as

(2) $$x_{n+1}=f\left(x_{n}\right)=\mu_{xn}\left(1-x\right),n=1,2,\ldots$$

where $x_{n\;}\in \left(0,1\right)$ and μ ∈(0,4) are known as the control parameter or bifurcation parameter.

Here,$\;x_{n}$ represents the system’s state at time $n.x_{n+1}$ indicates the following state, and n shows the discrete-time. By repeated iteration of *f*, a sequence of points $\left\{x_{n}\right\}\infty$ is increased, known as an orbit. The performance of the logistic map is sensitive to the value of *µ*. For μ ∈(3.574), the logistic map is chaotic [43]. Now, the diffusion algorithm key is chosen, for which the actual y, the primary iteration of the logistics, is with parameter μ. For different primary conditions, two logistic maps are utilized for executing the repetition operation. Moreover, the values of the state of two logistic maps are measured dynamically. With this operation, chaotic sequences are produced. The operation is as follows: place a grayscale image G with the size of m×n, the two-dimensional data matrix of R, turn R into the one-dimensional matrix with the length of m×n. Put R1 = {r_1_, r_2_…r_m×n_}, and put P_1_ = {p_1_, p_2_…p_m×n_} as the encrypted 1D matrix. The procedure of the encrypted algorithm will be as follows:

Step NO.1: In the first step, two chaotic sequences, *x* = {*x*_1_, *x*_2_… *x*_*m*×*n*_} are produced by two one-dimensional logistic maps. Place the two logistic maps system parameter as a primary value as *x*_1_(0) and *x*_2_(0), respectively.
Step NO.2: In the second step, for every iteration, compare *x*_1_(*i*), and *x*_2_(*i*), *i* = 1, 2, *m* × *n* and choose one that is numerically larger.
Step NO.3: In the next step, perform the Exclusive NOR (XNOR) operation for sequences produced by Step NO.2 with the original image’s pixels.
Step NO.4: In the last step, change the encrypted one-dimensional matrix, namely P, into a two-dimensional matrix. Set the size of this modified matrix to *m* × *n*. In this process, a two-dimensional data matrix R2 is generated. Thus, a diffused image is obtained.

### 2.3. Kinetics of Coupled Map Lattice

One of the most popular classes of models in the theory of space-time chaos is formed by the coupled map lattice (CML). Coupled map lattices are extensively applied to survey the dynamics of spatially prolonged logistic map systems. The CMLs are used in cryptography, physics, economics, steganography, and biology. They have a significant role in image encryption algorithms [44,45,46]. Then, we used a two-dimensional hyper-chaotic map CML to try pixel location. It can effectively and efficiently extend the keyspace. It increases the capability of anti-decryption. CML statement is as:

(3) $$x_{n+1}=1-a\left(x_{n}^{2}+y_{n}^{2}\right)$$

(4) $$y_{n+1}=-2a\left(1-2b\right)x_{n}y_{n}$$

The digital images possess the digital matrix features for scrambling the location of pixels; thus, considering a random image, the impact of confidentiality is accomplished. The procedure of the encrypted algorithm will be as follows:

Step NO.1: In the first step, the chaotic sequences *x*_1_, *x*_2_ = {*x*_1_, *x*_2_… *x_m_*} are produced with the length of m, and *y*_1_, *y*_2_ = {*y*_1_, *y*_2_… *y_n_*} with the length of n similar to CML chaos mapping.
Step NO.2: In the second step, *x*, *y* chaotic sequences are arranged in rising sequences, producing position sequences *w*_2_, *w*_3_.
Step NO.3: In the last step, the pixel confusion is performed, using *w*_2_, *w*_3_ as the row, and column sequences of the data matrix *R*.
  (5) $$R\left(i,1\right)=R\left(w_{2}\left(i,w_{3}\left(j\right)\right)\right).$$

### 2.4. Wavelet Transform

A valuable instrument for analyzing the signal’s frequency components is called the Fourier transform. Taking the Fourier to convert over the whole-time axis, it is impossible to determine the exact instant of increasing a specific frequency. The Fourier transform and the wavelet transform are the same with a completely various merit function. The wavelet transforms mainly aimed at only allowing changes by transforming the time extension rather than the shape. The main difference between these two is that the signal is decomposed by the Fourier transform into cosines and sine’s; however, the wavelet transform utilizes the functions localized in both the Fourier and real space. Commonly, the wavelet transform is stated as follows:

(6) $$f\left(a,b\right)=\int_{\infty }^{-\infty }f\left(x\right)\Psi^{*}\left(a,b\right).x.d\left(x\right)$$

in which * represents the complex conjugate symbol and function *φ* is a function which can be arbitrarily selected if it follows definite rules. The wavelet transform can have a signal into time, space, and frequency as independent space. It also focuses on the specific signal of any local details. Thus, further information can be extracted effectively from the signals much by wavelet transform.

Numerous types of wavelet transforms exist for particular purposes. We used continuous and discrete wavelet transforms to extract further information from the signals. Similar to the Fourier transform, inner products are used by the continuous wavelet transform for measuring the similarity between a signal and an analyzing function. Theoretical analysis is one of the areas for using a continuous wavelet transform. Within the particular realization on computers as a functional area of research, a continuous wavelet must be discretized [47,48,49,50]. Running the wavelet transformation through a discrete set of wavelet scales and translations following some determined rules is known as the discrete wavelet transform. The signal is decomposed by transforming into the mutually orthogonal group of wavelets as the necessary variation from the continuous wavelet transforms.

Moreover, the implementations for the discrete-time series are occasionally determined as the discrete-time continuous wavelet transforms. It is the most significant point to select the wavelet utilized for time-frequency decomposition. Through this selection, we can affect the frequency and time resolution of the results. This way cannot replace Wavelet Transformation (WT)’s basic features (low frequencies possess a wrong time resolution and true frequencies; higher frequencies possess a wrong frequency resolution and a good time). However, it is somehow possible to increment the total time resolution’s total frequency. It is straightly proportional to the utilized wavelet’s width in the Fourier and real space. Using the Morlet wavelet, we can presume high-frequency resolution as a very well-localized wavelet in frequencies. In reverse, utilizing a Derivative of Gaussian wavelet will lead to the right time localization but lower frequencies.

## 3. Proposed Algorithm

This section may be divided by subheadings. It should provide a concise and precise description of the experimental results, their interpretation, as well as the experimental conclusions that can be drawn.

**Figure 1:**
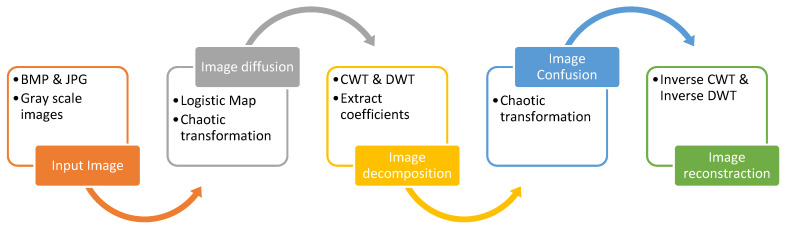
The proposed algorithm for image encryption: CWT: Continuous Wavelet Transform; DWT: Discrete Wavelet Transforms.

Figure 1 represents the proposed algorithm. The steps for implementing the suggested algorithm are:

Step NO.1: In the first step, a grayscale image G is arranged. The image’s size is set to *m* × *n*. Moreover, data matrix R is placed. By evaluating two logistic maps, a chaotic sequence is generated. Making XNOR with the primary image, the diffusion is terminated.
Step NO.2: In this step, for the diffused image in step NO.1, the wavelet decomposition is performed and then the wavelet coefficient is extracted, registered as ca1.
Step NO.3: Utilizing a two-dimensional hyper-chaotic map CML, the chaotic sequence is produced, and with ca1 established in step NO.2, the position confusion is performed.
Step NO.4: In the last step, the confused image can be rebuilt by wavelet. After all, the encrypted image is obtained. The inverse operations of the encryption are known as the decryption algorithm. System parameters and the primary value of the chaotic sequences in the image encryption and image decryption are consistent.

### 3.1. Encryption Assessments Metrics

We measured our cryptography scheme’s performance by selecting some basic parameters to assess the algorithm. Visual inspection is one of the main parameters for assessing the encrypted images [51,52,53]. The characteristic diffusion survey is another parameter [54,55] determined for judging the randomization algorithm. Through inspection, the deviation of a product from a definite set of features is determined. Human operators usually accomplish the inspection; nevertheless, machine vision is utilized for automating this procedure [56,57,58]. By the excellent diffusion of an algorithm, the association between the original image and the encrypted image becomes too complicated and cannot be predicted simply. Here, we studied the Peak Signal to Noise Ratio (PSNR) computation metrics, the association between the encrypted image and the key-image. Ultimately, we assessed the specification diffusion by calculating two parameters of the Unified Average Changing Intensity (UACI) and the Number of Pixels Change Rate (NPCR).

### 3.2. Peak Signal to Noise Ratio (PSNR)

Peak Signal to Noise Ratio (*PSNR*) is an engineering formulation determined through mean square error (*MSE*). It is generally utilized for image quality evaluation as follows [59]:

(7) $$PSNR=10\log\left(\frac{255^{2}}{MSE\left(f,f^{\prime }\right)}\right)$$

where *f*(*x*; *y*) and *f*’(*x*; *y*) denote the pixel values of m × n original and reconstructed images.

### 3.3. Number of Pixels Change Rate (NPCR)

Diffusion is represented by the number of the most essential parameters for judging the encryption algorithm randomization. NPCRs are used to examine the image encryption algorithm’s security. Considering *C*_1_ and *C*_2_ as the two images with *N* × *M* size, we defined an array, *D*, with the sizes similar to images *C*_1_ and *C*_2_ as:

(8) $$D\left(i,j\right)=\{{\;}_{1ifC_{1}\left(i,j\right)\neq C_{2}\left(i,j\right)}^{0ifC_{1}\left(i,j\right)=C_{2}\left(i,j\right)}$$

The *NPCR* determines the percentage of pixels within two different images, and it can be calculated as follows [31]:

(9) $$NPCR=\frac{\sum_{ij-1}^{N\times M}D\left(i,j\right)}{N\times M}\times 100\%$$

### 3.4. Unified Average Changing Intensity (UACI)

UACI determines the average intensity of the difference within the two encrypted images (C_1_ and C_2_), using the below expression [60]. It is applied to evaluate the encryption method’s strength. Its value is based on the image’s format and size [61,62]. Through UACI, the average variation in intensity between the ciphered and original images is assessed. The greatest UACI indicates that the suggested technique has resistance against various attacks. UACI is determined for the grayscale image of size *M* × *N* as follows:

(10) $$UACI=\frac{1}{N\times M}\left[\overset{N\times M}{\underset{ij-1}{\sum }}\frac{C_{1}\left(i,j\right)-C_{2}\left(i,j\right)}{MAX\left(C_{2}\right)}\right]\times 100\%$$

### 3.5. Correlation Coefficient

Digital Image Correlation (DIC) is a key and extensively utilized non-contact method to measure material deformation. In recent years, there has been a significant development in developing novel experimental DIC methods and in improving the relevant computational algorithms’ performance [63,64]. Thus, a relation is indicated among the same pixels of the encrypted and the original images as follows:

(11) $$NC=\frac{\sum m\times \sum n\left(A_{mn}-\overset{-}{A}\right)\left(B_{mn}-\overset{-}{B}\right)}{\sqrt{{\left(A_{mn}-\overset{-}{A}\right)}^{2}{\left(B_{mn}-\overset{-}{B}\right)}^{2}}}$$

where *A* and *B*, respectively, denote the original image and the encrypted one, as well as their means. The lower correlation coefficient value is optimal.

## 4. Experimental and Numerical Results

**Figure 2:**
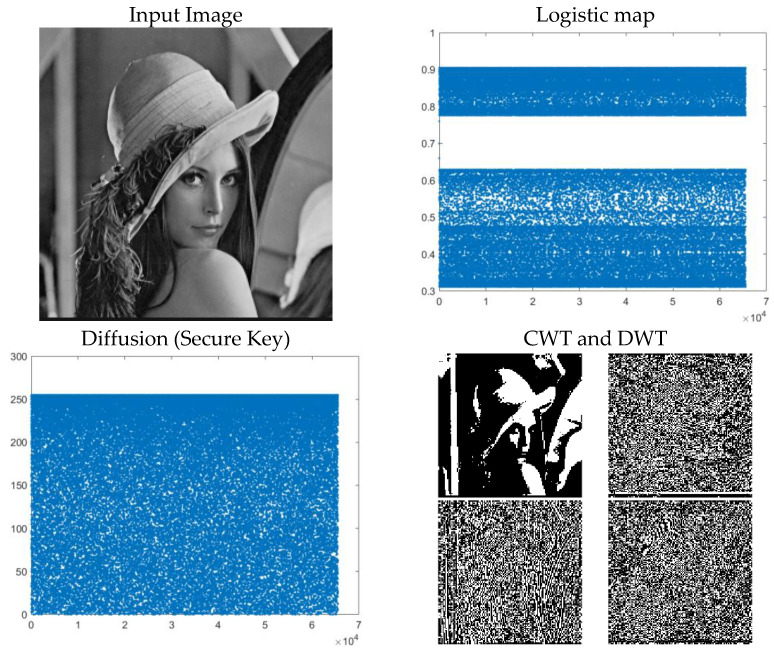

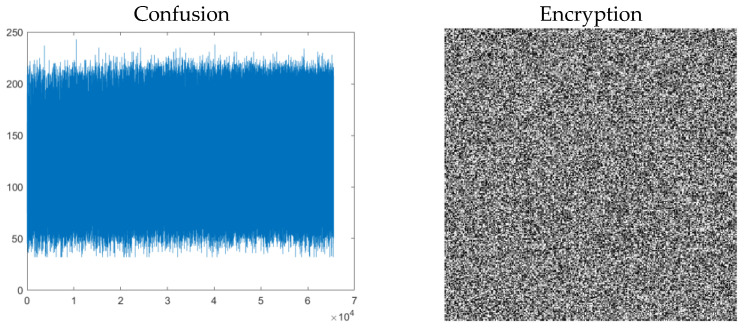
Results of the proposed method steps.

The results of the presented algorithm steps are indicated in Figure 2. In the first step, the input grayscale image with a size of m × n is imported. Based on Figure 2, a chaotic sequence is created with the two logistic maps used. Finally, in the diffusion step, the secure key is generated for encryption. For the encryption of the input image, the secure key must be inserted between the wavelet decomposition sub band. The sub bands of the DWT method are indicated in Figure 2. Upper to lower and left to right images in the DWT sub-bands are Low-Low, Low-High, High-Low and High-High sub bands. Utilizing a two-dimensional hyper-chaotic map CML, the chaotic sequence is produced and the confusion is performed. In the final step, the confused image is generated. Finally, the image consists of an encrypted matrix with the use of an input image and secure key.

Evaluating the suggested algorithm with numerical results indicates that this algorithm is robust. Numerical results for the proposed algorithm are displayed in Table 1.

**Figure 3:**
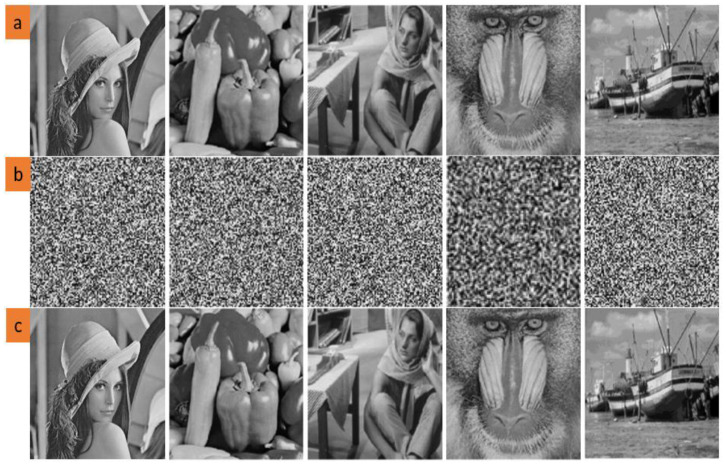
The visual results for applying the proposed algorithm to some images. (**a**) Original images. (**b**) Encrypted images. (**c**) Reconstructed images.

First, the diffusion operation is performed for the encryption of the primary image (original image). The primary key value is taken from Table 2; then, the confusion operation is performed while taking the primary key value from Table 2. The findings of encryption are the same as the noise (Figure 3). No information on the original image is acquired from the encrypted image. A decrypted image is acquired via the key for decrypting the encrypted image, followed by diffusion and confusion operations (Figure 3, decrypted image).

### 4.1. Histogram Analysis

**Figure 4:**
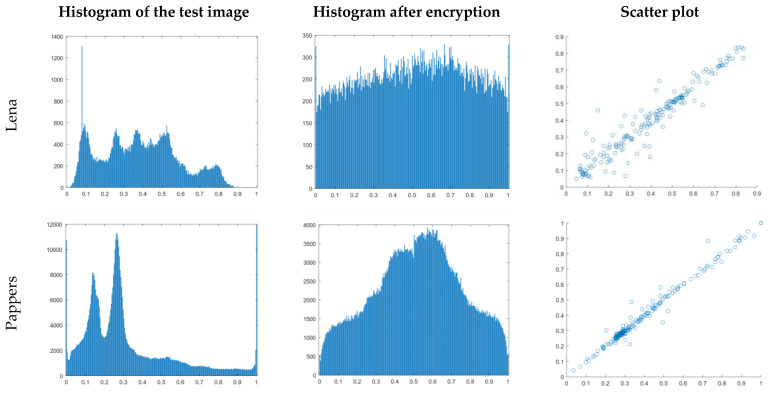

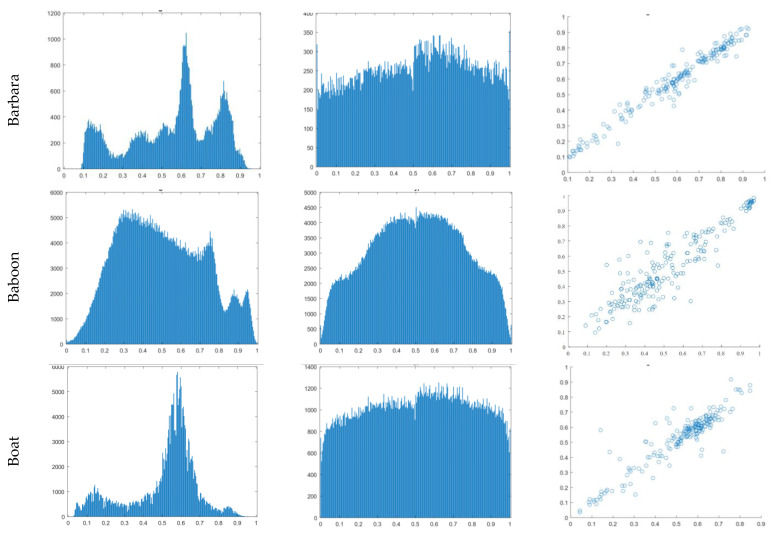
The histogram analysis for original Lena image.

The first test is the histogram analysis of the encrypted, decrypted, and original images. Here, respective images’ image histograms represent the vast differences between encrypted and original images, while they are the same. With the evaluation of the histogram of the test image and histogram after encryption, it is observed that the encrypted image is distributed uniformly in the entire interval of the histogram. Therefore, the original image’s distribution regularity is covered. Hence, the encryption is implemented effectively (see Figure 4).

### 4.2. Complexity

Using the proposed algorithm, by encrypting an image A of M × N, the algorithm using the chaotic map should produce an M × N number of random numbers R1. Hence, the complexity to produce M × N numbers of the random number is O (n). By increasing the time using the same chaotic map, the algorithm should produce a random chaos sequence of M × N bits. Hence, the complexity is repeated to produce M × N numbers of random bits as O (n). Afterward, it builds series (M × N) of chaos sequence additions or subtractions with a complexity of O (n). Lastly, it builds a chain XOR of operations as O (n). Thus, the algorithm’s whole complexity is O (n).

### 4.3. Robustness

**Figure 5:**
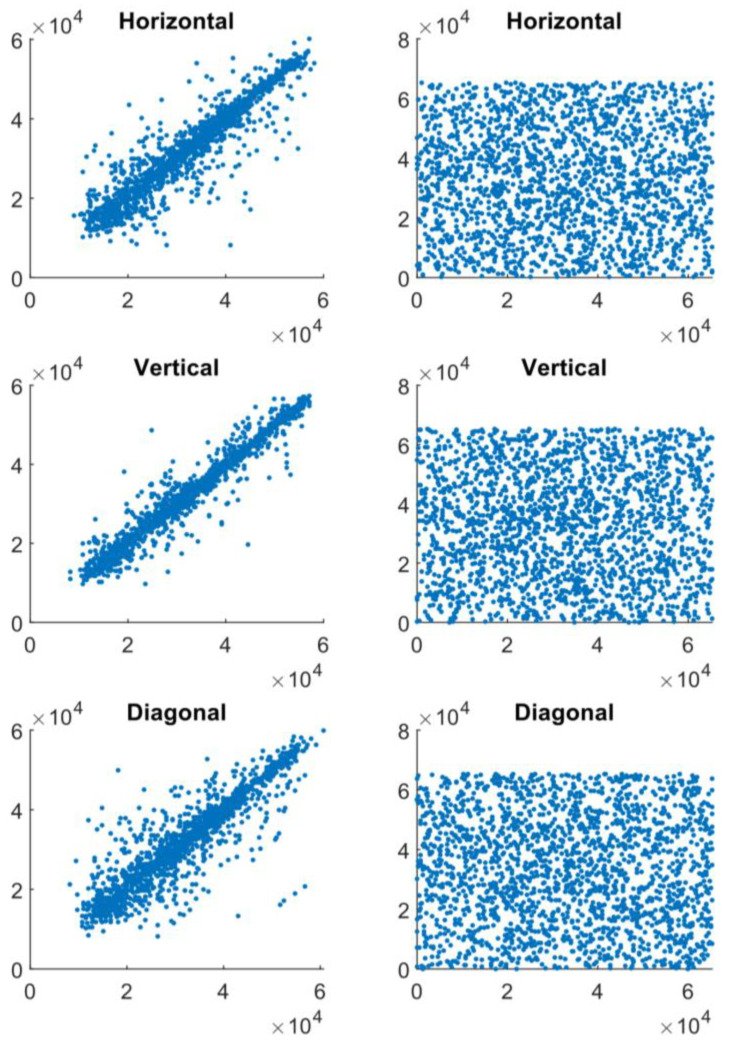
Plots of pixel horizontal, vertical and diagonal, correlation for input (**Left**) and encrypted images (**Right**) in Lena image.

We evaluated the correlation between two vertically, two horizontally, and two diagonally adjacent pixels in the input image and encrypted image in addition to the histogram analysis in Figure 5. The values of two adjacent pixels in the image are represented by the x- and y-axes. In both the input and cipher images, Figure 5 depicts the correlation distribution of two horizontally adjacent pixels. Both the plain image and the cipher image have correlation coefficients of 0.99 and 0.02, respectively. The diagonal and vertical directions both yield similar results. The simple picture has a high correlation of two neighboring pixels.

To evaluate the proposed approach’s robustness, the test images are tested against four types of image processing attacks: rotation, Gaussian noise, median filtration, and histogram equalization. The results show that the proposed design is associated with higher robustness and normalized correlation. Based on the results, input attack does not affect image encryption and decryption. Regarding the Normalized Correlation (NC) value for different types of images, median filter, rotation, and Gaussian noise have higher NC values. This means that the robustness of the presented method resists these types of attacks. However, the impact of histogram equalization is remarkable (see Table 3). 

## 5. Discussion

Here, a novel algorithm was presented for image encryption oriented by chaos. The provided algorithm in this paper has the following benefits in comparison with other chaos-based algorithms. The chaotic sequence system structure is more complicated than the low-dimensional one, producing an integration of multivariate or univariate chaotic sequences [65]. This algorithm uses two one-dimensional chaotic systems. One-dimensional chaotic maps became an attractive field with the first detection of the Logistic Map in 1976. A very simple map by May [66] indicated that chaotic behavior could be exhibited using even a very simple nonlinear equation (a one-dimensional quadratic equation). Hence, our primary objective, as well as the ratio of taking this kind of map, is to discover a novel discrete time-series, the same as the logistic map exhibiting chaotic performance for unique parameters with elementary equations. The proposed method, in comparison with a low-dimensional chaotic sequence, is very secure for generating the chaotic sequence.

Moreover, compared to the high-dimensional chaotic sequence, this algorithm has a smaller calculation burden. The reason is that through the chaos model reconstruction, a low-dimensional chaotic sequence is simply attacked. Low-dimensional chaos was utilized for image encryption; however, its chaotic orbit was simple and might be simply predicted through the methods such as regression mapping, nonlinear prediction, and phase space reconstruction. Thus, the image encryption scheme utilizing low-dimensional chaos is simply exposed to the attacks [67]. However, the proposed method can transform the dynamic performance of the original chaotic system through dynamic comparison while completely resisting the model reconstruction attack, therefore developing its security. Compared to a high-dimensional chaotic sequence, the one-dimensional sequence has a lower amount of calculation. The reason is that the low-dimensional chaotic system is usually demonstrated with an algebraic equation, and it has a rapid solution. However, the high-dimensional chaotic system is a complex differential equation with relatively more considerable complexity and calculation burden. Utilizing the high-dimensional chaotic systems in some image encryption algorithms, the encryption procedure was straightforward. Moreover, the encryption algorithm was not sensitive to the secret keys and plain image alterations exposed to selective plaintext attacks or plaintext attacks [68]. By performing confusion encryption after diffusion encryption, we can develop the capability against the confusion encryption attack. Since the diffusion outcome becomes hidden by confusion encryption, the cipher is impractical through gathering the specific image. Classical encryption is commonly used just in the frequency-domain or air-domain. Our research, air-domain, and frequency-domain simultaneously perform encryption to improve the effects of encryption and enhance the encryption intensity.

Moreover, it is difficult to break in the frequency-domain or air-domain. Since the two-dimensional hyper-chaotic map is utilized in confusion encryption, there is a more considerable calculation burden for this algorithm. The findings obtained from the experimental values for the various standard images obtained by applying some existing methods, including our presented model, are shown in Table 4. These findings indicate that our approach is highly vulnerable to the alteration of the plain image bit, thereby making void differential attacks. In the presented model, input images consist of a 2D matrix of grayscale images. The main advantage of these types of images is to reduce both process time and storage volume. However, there are some disadvantages. Sometimes, the encryption should be implemented on a color image or video. In color pictures, video files, and voice files, encryption plays an important role. Therefore, the main limitation of this method is incompatibility with other types of files. For future hypotheses and research, we suggest extending the presented method and testing on other types of presentative files.

The suggested scheme’s encrypted picture has a uniform histogram, a near-to-zero correlation coefficient, and entropy close to the full entropy. All of this shows that the scheme can withstand statistical attacks very well. The NPCR scores are appropriate for avoiding differential attacks, and the UACI scores are similar to the optimal result. Furthermore, the processing time for encryption and decryption is strictly proportional to the magnitude of the original image’s correlation coefficient. A simple image with a lower correlation coefficient takes less time to encrypt and decode, and vice versa. The proposed scheme has a broad chaotic regime for a wide variety of parameters, provides good security, and can withstand typical attacks, according to the dynamical analysis and assessment findings.

## 6. Conclusions

Recently, various chaos-based image cryptosystems have been presented. The present work deals with a chaotic-based algorithm using characteristics of the chaotic map and wavelet transform. The encryption process in this algorithm includes two stages. At first, we performed the image diffusion operation. Moreover, by performing the wavelet transform, the calculation amount in confusion was considerably reduced by hyper-chaotic sequences. The simulation results with the standard metrics show that the proposed algorithm has a high dependence on keys. This algorithm includes a decent encryption effect. Moreover, it can resist noise and cut attacks. We have tested the presented method for Lena, Peppers, Barbara, Baboon, and Boat Images from benchmark MATLAB test images. Moreover, the histograms of both input images and encrypted images are depicted. In addition, the encryption performance analysis criteria such as PSNR, NPCR, UACI and NC are recorded. Based on the results, the correlation value for Lena, Peppers, Barbara, Baboon, and Boat is 95.48%, 99.64%, 98.09%, 91.37% and 90.01%, respectively. To evaluate the proposed approach’s robustness, the test images are tested against four types of image processing attacks: rotation, Gaussian noise, median filtration, and histogram equalization. The results show that the proposed design is associated with higher robustness and normalized correlation. Based on the results, input attack does not affect image encryption and decryption. Regarding the NC value for different types of images, median filter, rotation, and Gaussian noise have higher NC values. It means that the robustness of the presented method resists these types of attacks. However, the impact of histogram equalization is remarkable. For future work, we suggested implementing the presented method for other types of files such as voice, video, and color 3D images. 

## Figures and Tables

**Table 1:** The numerical results of the proposed algorithm.

Image	Type of Image	PSNR	NPCR	UACI	NC
Lena Image	Jpg	42.612	99.757	33.120	0.9548
Peppers Image	Jpg	39.220	99.787	33.621	0.9934
Barbara Image	Jpg	36.841	99.626	33.126	0.9809
Baboon Image	Jpg	39.134	99.881	33.415	0.9137
Boat Image	Jpg	38.223	99.625	33.671	0.9001

**Table 2:** Key initial value for diffusion and confusion operations.

x1(1)	x2(1)	µ 1	µ 2
0.5	0.5	4	3.9
x3(1)	y3(1)	µ 1	µ 2
0.3	0.3	4	3.9

**Table 3:** The NC value of the proposed algorithm with different types of attack.

Image	Median Filter	Histogram Equalization	Rotation	Gaussian Noise
Lena Image	0.984	0.987	0.999	0.999
Peppers Image	0.704	0.280	0.923	0.964
Barbara Image	0.914	0.497	0.980	0.991
Baboon Image	0.960	0.629	0.991	0.996
Boat Image	0.976	0.746	0.995	0.998

**Table 4:** The numerical results of the proposed algorithm in comparison with state-of-the-art methods.

Reference	Image	NPCR	UACI
Presented model	Lena Image	99.757	33.120
Presented model	Peppers Image	99.787	33.621
Presented model	Barbara Image	99.626	33.126
Presented model	Baboon Image	99.881	33.415
Presented model	Boat Image	99.625	33.671
Amina et al. [69]	Lena Image	99.646	33.625
Amina et al. [69]	Peppers Image	99.632	33.507
Amina et al. [69]	Baboon Image	99.602	33.629
Yavuz et al. [70]	Lena Image	99.620	33.410
Zhang and Zhao [71]	Lena Image	99.605	33.411
Assad and Farajallah [72]	Lena Image	99.607	33.463
Assad and Farajallah [72]	Boat Image	99.615	33.465
Kari et al. [38]	Lena Image	99.646	33.625
Kari et al. [38]	Peppers Image	99.713	33.541
Kari et al. [38]	Baboon Image	99.623	33.416
Kari et al. [38]	Boat Image	99.619	33.556

## Data Availability

In this study we used MATLAB benchmark images for academic studies. Available in MATLAB software samples.
